# Mining TCGA Data for Key Biomarkers Related to Immune Microenvironment in Endometrial cancer by Immune Score and Weighted Correlation Network Analysis

**DOI:** 10.3389/fmolb.2021.645388

**Published:** 2021-03-26

**Authors:** Chengbin Guo, Yuqin Tang, Yongqiang Zhang, Gen Li

**Affiliations:** ^1^ Guangzhou Women and Children’s Medical Center, Guangzhou Medical University, Guangzhou, China; ^2^ School of Basic Medical Sciences, Chengdu University of Traditional Chinese Medicine, Chengdu, China; ^3^ Molecular Medicine Center, West China Hospital, Sichuan University, Chengdu, China; ^4^ West China School of Medicine, West China Hospital, Sichuan University, Chengdu, China

**Keywords:** endometrial cancer, immune microenvironment, biomarker, Estimate, CIBERSORT, WCGNA

## Abstract

**Background:** Endometrial cancer (EC) is one of the most lethal gynecological cancers around the world. The aim of this study is to identify the potential immune microenvironment-related biomarkers associated with the prognosis for EC.

**Methods:** RNA-seq data and clinical information of EC patients were derived from The *Cancer* Genome Atlas (TCGA). The immune score of each EC sample was obtained by ESTIMATE algorithm. Weighted gene co-expression network analysis (WGCNA) was used to identify the interesting module and potential key genes concerning the immune score. The expression patterns of the key genes were then verified *via* the GEPIA database. Finally, CIBERSORT was applied to evaluate the relative abundances of 22 immune cell types in EC.

**Results:** Immune scores were significantly associated with tumor grade and histology of EC, and high immune scores may exert a protective influence on the survival outcome for EC. WGCNA indicated that the black module was significantly correlated with the immune score. Function analysis revealed it mainly involved in those terms related to immune regulation and inflammatory response. Moreover, 11 key genes (APOL3, C10orf54, CLEC2B, GIMAP1, GIMAP4, GIMAP6, GIMAP7, GIMAP8, GYPC, IFFO1, TAGAP) were identified from the black module, validated by the GEPIA database, and revealed strong correlations with infiltration levels of multiple immune cell types, as was the prognosis of EC.

**Conclusion:** In this study, 11 key genes showed abnormal expressions and strong correlations with immune infiltration in EC, most of which were significantly associated with the prognosis of EC. These findings made them promising therapeutic targets for the treatment of EC.

## Introduction

Endometrial cancer (EC) is one of the most common gynecologic malignancies and represents the leading cause of morbidity and mortality among women worldwide (Ventriglia et al., 2017). Importantly, the incidence of EC is rising in the United States and more than 20 other countries (Lortet-Tieulent et al., 2018). In 2020, it occurred 65,620 new cases and caused 12,590 deaths in the United States (Siegel et al., 2020). The 5-years overall survival rate for early stage is about 81% while that for advanced stages (IVA and IVB) is approximately 15% (Siegel et al., 2018).

EC is histologically classified into several subtypes, including endometrioid endometrial adenocarcinoma (EEC), serous endometrial adenocarcinoma (ESC), mixed serous and endometrioid (MSE), clear cell, and malignant mixed Mullerian tumors (MMMT) (Gaber et al., 2016; Urick and Bell, 2019). EEC is the most common histology, representing about 75% of all endometrial cancers, followed by ESC (1–5%) and clear cell (1–5%) (Murali et al., 2014). While the endometrioid subtype can be high or low grade, the other histological subtypes, especially ESC and clear cell, are generally high in grade with worse prognoses (Sorosky, 2012). EEC is characteristically driven with estrogen receptors (ER) and progesterone receptors (PR) and thanks to its early symptoms like abnormal uterine bleeding, EEC is usually diagnosed early (Lax et al., 1998).

**FIGURE 1:**
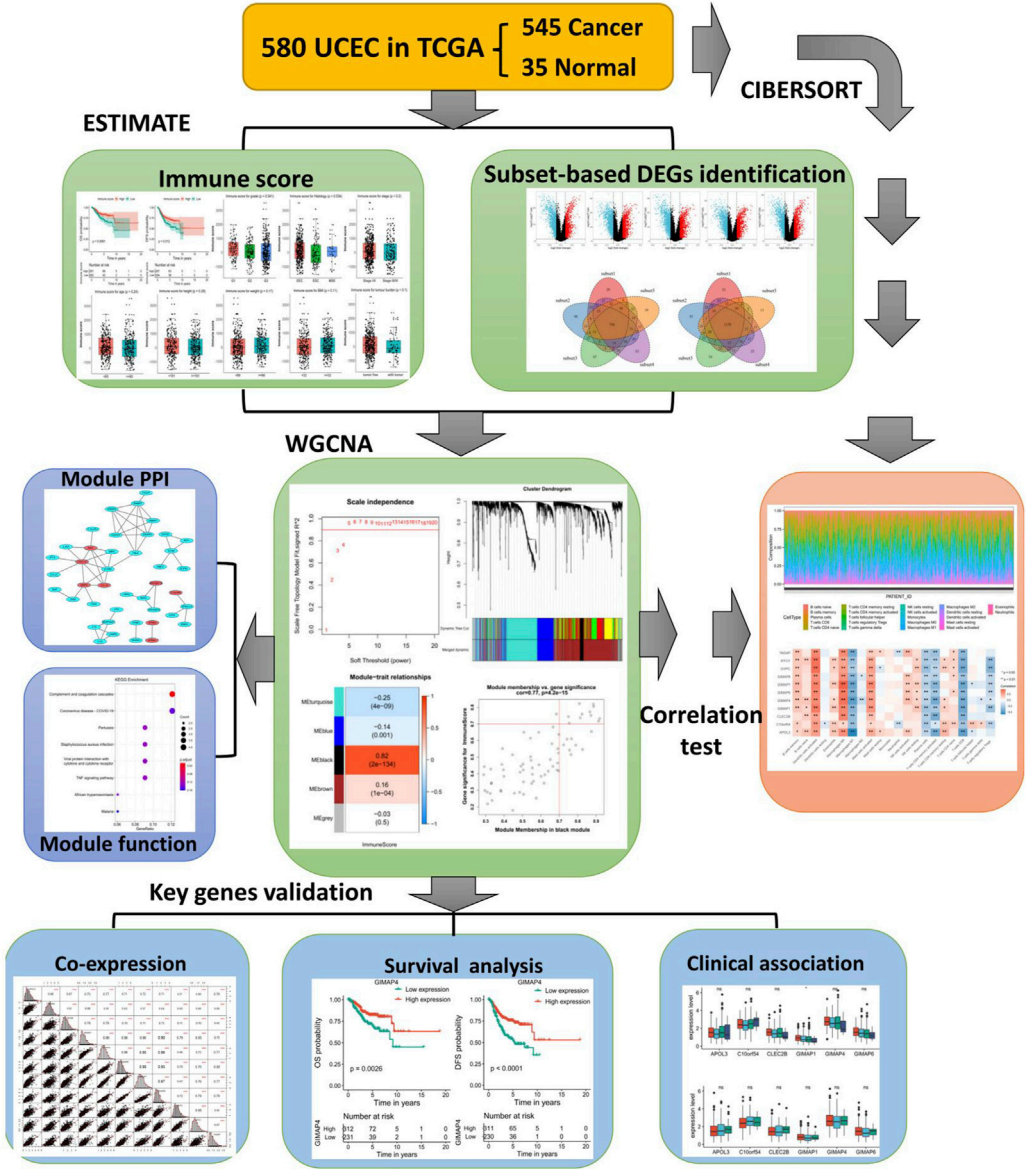
Flowchart of this study.

Despite remarkable advances of novel therapies, such as chemotherapy and radiotherapy, surgical resection with hysterectomy and bilateral salpingo-oophorectomy remains the primary and standard clinical intervention for EC patients, and the postoperative 5-years survival rate is till unfavorable, posing a huge threat to women’s lives. Immunotherapy, based on the concept of stimulating the endogenous immune response against tumor cells, has become a dependable clinical strategy in cancer treatment. For example, the agents blocking PD1/PD-L1 have exhibited impressive effects on lung, renal cancer, and melanoma (Terme et al., 2011). It has been found that endometrial tumor cells can activate PD-1 signaling and the PD1/PD-L1 expression levels in EC (40%–80% in EEC, 10%–68% in ESC, and 23%–69% in clear cell subtypes) represent the highest expression among gynecologic cancers, thus holding great promise for EC treatment (Brahmer et al., 2012; Vanderstraeten et al., 2015). In this study, we used several algorithms including ESTIMATE and CIBERSORT to assess the immune scores and immune infiltration in EC and identified 11 potential immune therapeutic targets involved in the regulation of the immune microenvironment (IME) of EC, providing candidate prognostic biomarkers for EC. The workflow for this study is shown in Figure 1.

## Materials and Methods

### Data Acquisition, Immune Score Generation, and Clinical Relationship

We retrospectively collected the gene expression profilings of 545 endometrial adenocarcinoma (cases were enrolled according to basic clinical information including gender, age, subtype, grade, and stage) and 35 normal tissue samples from the TCGA database (https://portal.gdc.cancer.gov/). The corresponding clinicopathological parameters including age, height, weight, BMI, histology, TNM stage, tumor grade, tumor burden, and survival data were also obtained. For data preprocessing, gene names were transformed to official gene symbols with Perl language, and the only genes with non-zero expression values in at least half of the sample type were kept. The immune score of each tumor sample was calculated with the ESTIMATE algorithm using the estimate package (Yoshihara et al., 2013) based on R language software (version 3.6.0). Afterward, the immune scores were compared between different subgroups according to clinicopathological parameters with the Wilcox test. To evaluate the prognostic associations, Kaplan-Meier plots for overall survival (OS) or disease-free survival (DFS) in high- or low-immune score groups were depicted based on the optimized immune score value (−1.322856 and −11.99095 for OS and DFS, respectively) of each patient, with a log-rank test for statistical significance. Besides, given that the IME might be correlated with tumor stemness (Yi et al., 2020), we also obtained the available mRNA expression-based stem index (mRNAsi) of 528 EC patients as previously reported (Malta et al., 2018), following with the exploration of the spearman correlation between immune score and miRNAsi.

### Screening the Differentially Expressed Genes (DEGs) in EC

DEGs analysis between EC and normal tissues was performed by using the “limma” package (Ritchie et al., 2015), with the criteria of adj.P.Val <0.01 and | logFC| >1. Because the sample size of the tumor group was much larger than that of the normal group (545 vs 35), we adopted the subset-based strategy to balance the samples. Specifically, we randomly generate a subset of 50 EC tumor samples from the EC group five times without repetition, yielding a ratio of about 1.4:1 for tumor and normal samples. DEGs were screened by comparing the expression profile of each tumor subset and that of the normal tissue group. Venn diagrams were plotted to get the common DEGs by the five independent subset-based analyses.

### Weighted Gene Co-expression Network Analysis

Weighted gene co-expression network analysis (WGCNA) is a systematic algorithm to cluster highly correlated genes and to identify significant modules or key genes that are associated with a certain phenotype. In the current study, we utilized the WGCNA package (Langfelder and Horvath, 2008) to construct a gene co-expression network of common DEGs. In brief, sample clustering was conducted with the average linkage method to recognize and remove outlier samples, followed by the selection of the appropriate soft thresholding power (β) to achieve a scale-free topology fitting index of >0.9. Then the adjacency was transformed into a topological overlap matrix (TOM) and the corresponding dissimilarity matrix (1-TOM), which was further used to implement the gene clustering dendrogram with the minimum module of 30. Highly similar dynamic modules were merged into larger ones at the cutline of 0.2. Pearson correlation analysis was carried out to evaluate the relationships between modules and the immune score. The most significant module was identified and the gene significance (GS) and module membership (MM) were calculated. Key genes were defined as those with the GS > 0.7 and MM > 0.7 in this module.

### PPI Network Construction

A protein-protein interaction network of the identified module was constructed by STRING database (https://www.string-db.org) version 11.0 using the median confidence (combined score >0.4) and visualized by Cytoscape software (version 3.2.1). The network topology including node degree was investigated by the cytohubba application.

### Function Enrichment Analysis

To explore the involved biological functions and pathways of the significant module, we conducted Gene Ontology (GO) and Kyoto Encyclopedia of Genes and Genomes (KEGG) analysis with the “clusterProfiler” R package. The significant terms were defined as those with a p.adjust value of <0.05.

### Key Genes Validation

GEPIA is a widely-used web tool for data mining and visualization of the RNA sequencing expression data from the TCGA and the GTEx projects (Tang et al., 2017). In this study, we applied GEPIA to validate the differential expression of the key genes identified by WGCNA. The default parameters (|Log2FC| Cutoff of 1, and *p*-value Cutoff of 0.01, and log2 (TPM +1) for log-scale) were employed. Match TCGA normal and GTEx data were combined as the normal group (n = 91). Besides, the associations of the key genes and tumor grade or tumor histology were further explored, and the correlation matrix of the key genes was generated.

### Survival Analysis of the Key Genes

In order to evaluate the prognostic values of the key genes, we carried out the Kaplan-Meier survival analysis for OS (n = 543) and DFS (n = 541) with the aid of the EC samples from TCGA. For each key gene, patients were assigned to high- or low-expression groups according to the optimized immune score value of each patient. Statistical significance was measured by the log-rank test.

### Estimation of the Immune Cell Landscape

CIBERSORT, an analytical tool providing an estimation of the abundances of member cell types *via* gene expression data (Chen et al., 2018), was introduced to evaluate the tumor immune infiltration levels of EC. The relative proportion of 22 tumor immune cell types including B cells naïve, Plasma cells, B cells memory, T cells CD8, T cells CD4 naïve, T cells CD4 memory activated, T cells CD4 memory resting, T cells regulatory (Tregs), T cells follicular helper, T cells gamma delta, Monocytes, NK cells activated, NK cells resting, Macrophages M0, Macrophages M1, Macrophages M2, Dendritic cells activated, Dendritic cells resting, Mast cells activated, Mast cells resting, Eosinophils and Neutrophils were computed. Moreover, the relationship between key genes and each of the immune cell types was investigated.

## Results

### Correlations Between Immune Score and Clinical Characteristics in EC

**FIGURE 2:**
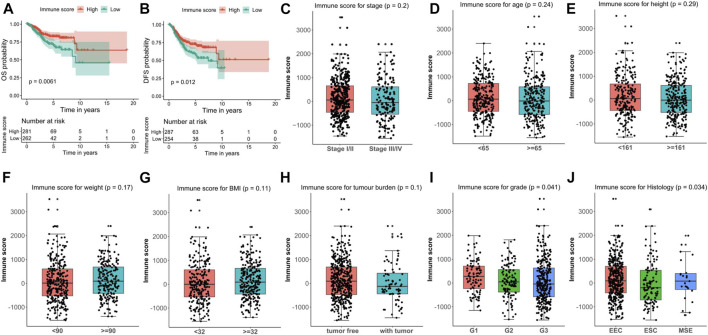
Correlation between immune score and clinical characteristics in endometrial carcinoma (EC). **(A, B)** Kaplan–Meier survival plots of immune score for OS **(A)** and DFS **(B)** for EC patients. **(C**–**J)** Associations of immune score and clinical characteristics including stage **(C)**, age **(D)**, height **(E)**, weight **(F)**, BMI **(G)**, tumor burden **(H)**, grade **(I)**, and histology **(J)**. OS, overall survival. DFS, disease free survival.

For the assessment of the correlations between immune scores and clinical outcomes, the high immune score group of EC patients showed a significantly superior overall survival (OS) or disease-free survival (DFS) than that of the low immune score group (*p* = 0.0061 for OS and *p* = 0.012 for DFS, respectively) (Figures 2A,B; Supplementary Figure S1). To investigate the relevance to clinical variables, 545 EC patients were classified by grade, histology, stage, age, height, weight, BMI, and tumor burden. Consequently, among these clinical characteristics, no significant associations were observed between immune score and tumor stage (*p* = 0.2), age (*p* = 0.24), height (*p* = 0.29), weight (*p* = 0.17), BMI (*p* = 0.11) or tumor burden (*p* = 0.1) (Figures 2C–H). However, the immune score was significantly associated with tumor grade (*p* = 0.041) and histology (*p* = 0.034) (Figures 2I,J). Specifically, for tumor grade, grade G3 (poorly differentiated) had a significantly lower immune score than G1 and G2. In terms of tumor histology, it seemed the ESC subtype has the lowest immune score. Besides, according to the Pearson correlation analysis, a significantly negative correlation might exist between the mRNA stemness index (mRNAsi) and the IME in EC patients (Supplementary Figure S2).

### Screening of DEGs in EC

**FIGURE 3:**
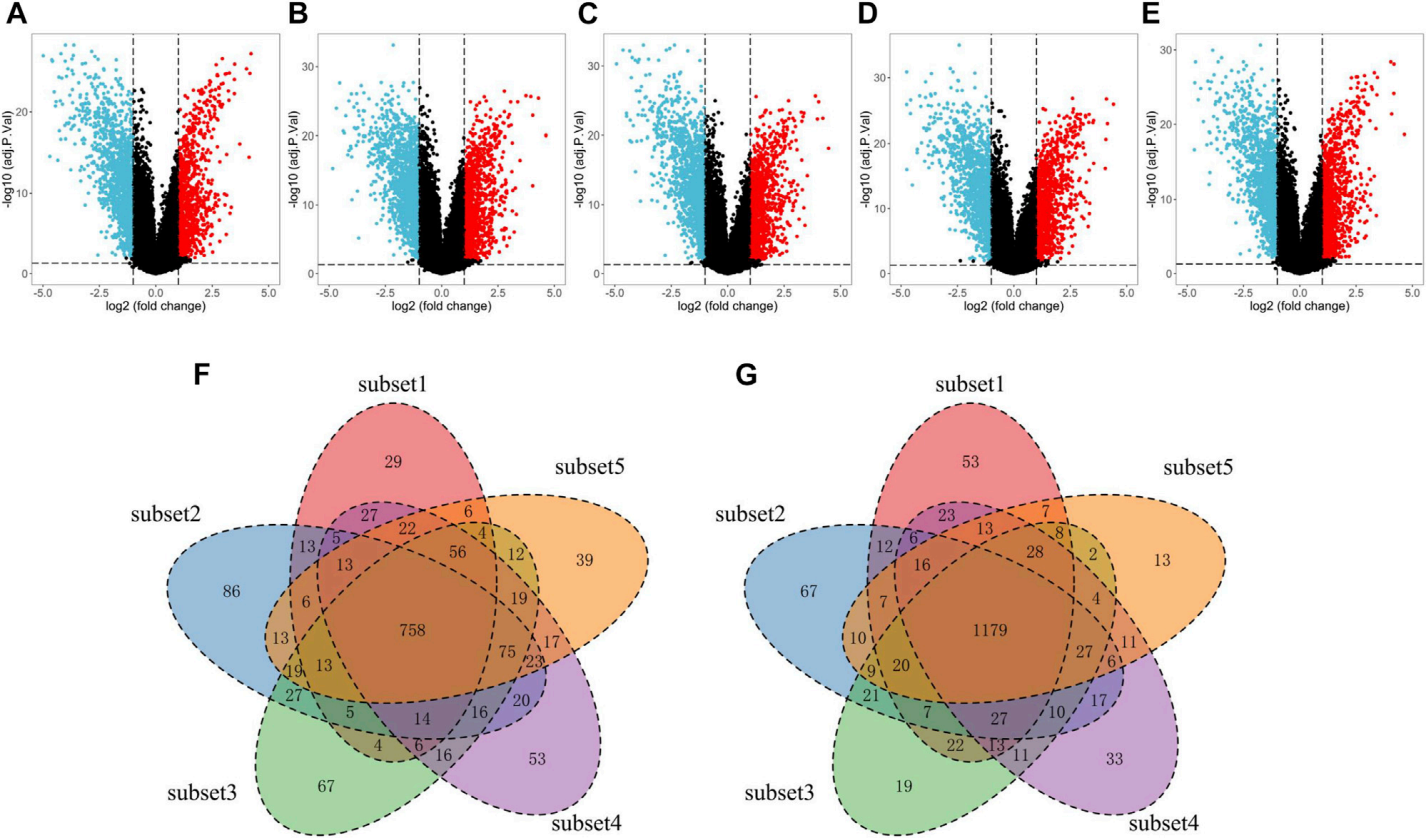
Screening of the DEGs in EC tissues compared with normal tissues with the subset-based approach using the TCGA-UCEC dataset. **(A–E)** Volcano plots showing the DEGs identified from five subsets of the TCGA-UCEC dataset. Red dots represent the upregulated genes, while blue dots represent the downregulated genes. **(F–G)** Venn diagrams showing the common upregulated genes **(F)** and common down-regulated genes **(G)** shared by the five subsets-based DEGs. DEGs, differentially expressed genes. EC, endometrial cancer.

The significant correlations between immune score and clinical characteristics or survival outcomes prompt us to hypothesize that oncogenes or tumor suppressor genes might be linked to different immune microenvironments of EC. Therefore, we screened the DEGs between EC and normal samples with the subset-based approach using the TCGA-UCEC dataset. First, we randomly subsampled 50 EC samples from the tumor group five times. Second, we obtained the DEGs between EC samples from each subset and the 35 normal samples, respectively (Figures 3A–E). As a result, 2,421 (980 up-regulated and 1,441 down-regulated), 2,545 (1,105 up-regulated and 1,440 down-regulated), 2,516 (1,110 up-regulated and 1,406 down-regulated), 2,563 (1,140 up-regulated and 1,423 down-regulated), and 2,454 (1,094 up-regulated and 1,359 down-regulated) DEGs were discerned in subset 1, 2, 3, 4, and 5, respectively. We next intersected the DEGs from all five subsets using two Venn diagrams, including a total of 758 common up-regulated genes and 1,179 common down-regulated in EC (Figures 3F,G).

### Co-Expression Network of DEGs in EC

**FIGURE 4:**
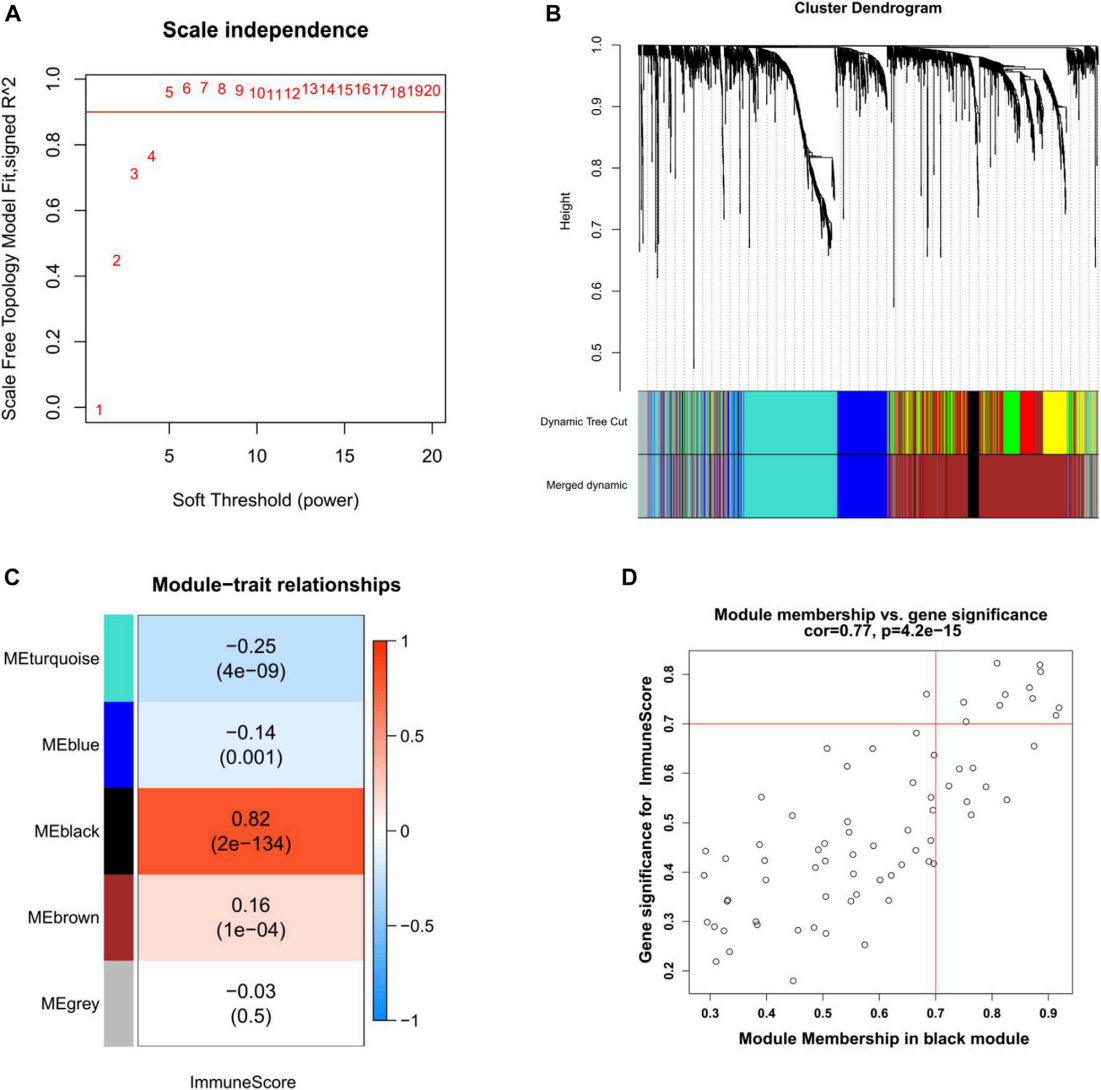
Weighted gene co-expression network analysis of EC. **(A)** Selection of the optimal soft-thresholding power for the scale-free network. **(B)** DEGs dendrogram using the dissimilarity measure (1-TOM). **(C)** Correlation analysis of module eigengenes and immune score. Pearson’s correlation coefficient and the corresponding *p*-value are shown. **(D)** Scatter plot of the black module showing the relationship between GS and MM. Key genes are indicated in the upper-right corner with the threshold of GS > 0.7 and MM > 0.7. TOM, topological overlap matrix. GS, gene significance. MM, module membership.

WGCNA was used to construct a co-expression network of DEGs in EC. Seven outlier samples were removed prior to network construction. The optimal soft-thresholding power of 5 (scale-free R2 = 0.96) was picked to ensure the scale-free topology (Figure 4A). A total of seven modules were screened out after merging dynamic modules with the Diss Thres of 0.2 (Figure 4B). Then, we focused on the most correlated module with the immune score in EC by computing the Pearson correlation coefficient (PCC) and corresponding *p*-value. As Figure 4C indicated, the black module was found to be significantly positive with immune score (PCC = 0.82, *P* = 2E-134), including 71 DEGs in EC. With the thresholds of GS > 0.7 and MM > 0.7 to further narrow down the range of candidate key genes, 11 DEGs were finally identified for the subsequent analysis (Figure 4D).

### The PPI Network of the Black Module

**FIGURE 5:**
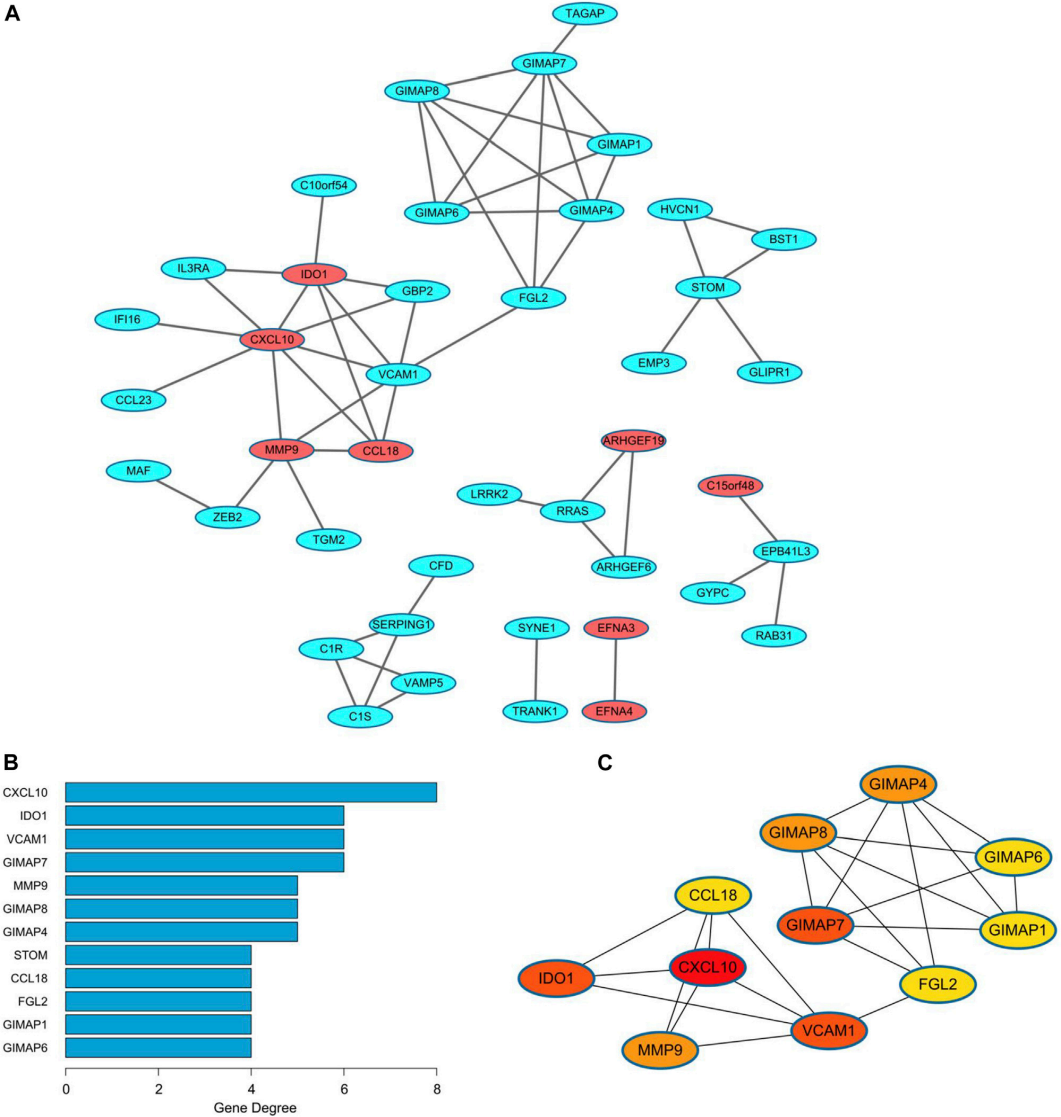
PPI network of the black module in endometrial cancer. **(A)** The PPI network consisting of 42 genes from the black module. Red nodes represent the upregulated genes, while the blue nodes represent the downregulated genes. **(B)** The top 12 nodes with the highest degree in the network. **(C)** The significant cluster formed by the top 11 nodes with most neighbor genes. PPI, protein-protein interaction.

A PPI network was built to analyze the black module explored above, containing a total of 42 DEGs (Figure 5A). There were totally eight up-regulated genes (red) and 34 down-regulated genes (blue) in the PPI network. Interestingly, several members of the GTPase IMAP family, i.e., GIMAP1, GIMAP4, GIMAP6, GIMAP7, and GIMAP8 formed a close submodule. For network topology, the top 12 hub nodes in the entire PPI network with their degrees were visualized in Figure 5B, the top 11 of which were closely connected and used to construct a sub-network (Figure 5C). The most important protein is CXCL10 (8 edges) in EC.

### Function Analysis of the Black Module

**FIGURE 6:**
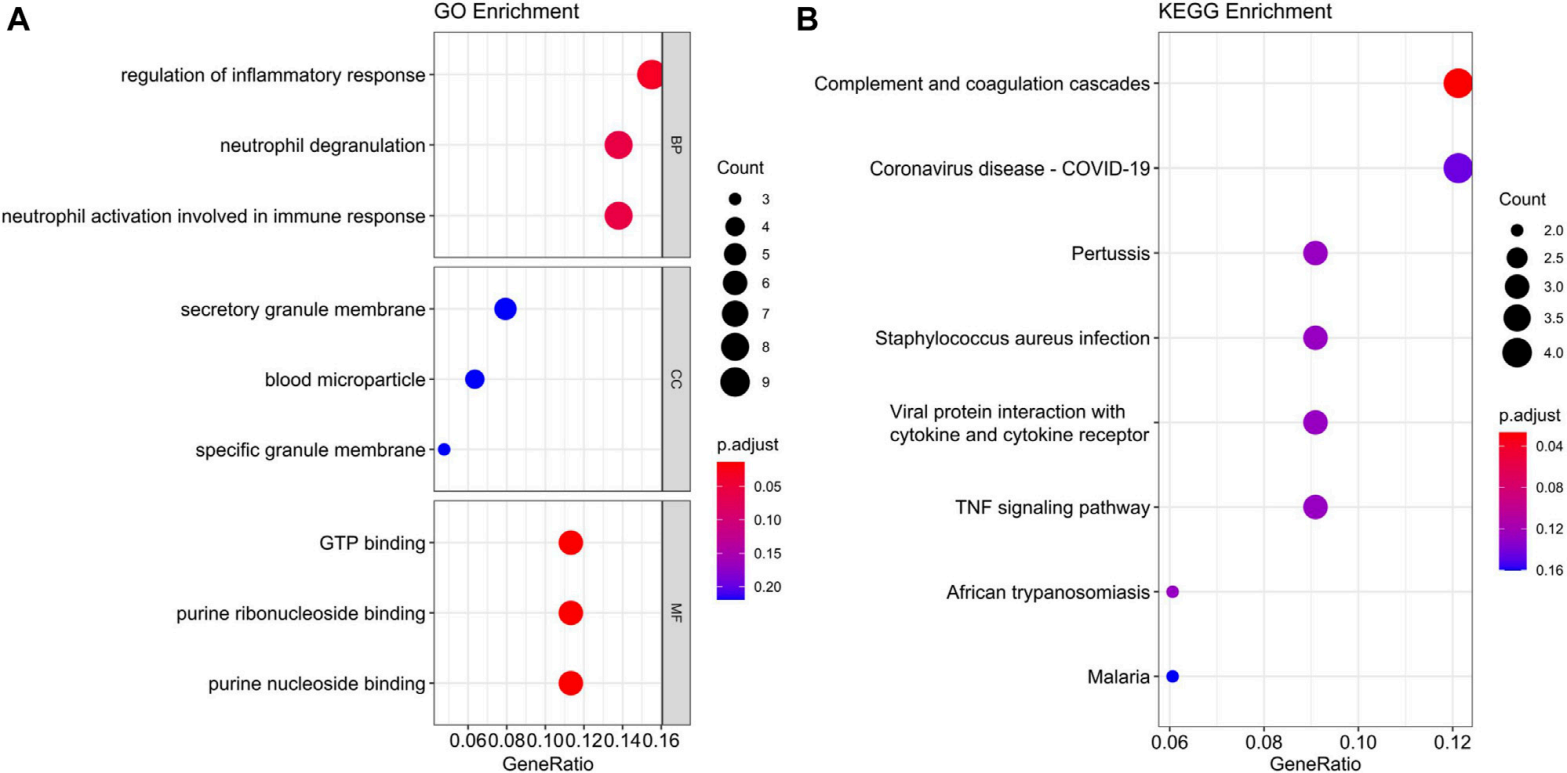
GO **(A)** and KEGG **(B)** enrichment analysis of the black module in endometrial cancer. BP, biological process. CC, cellular component. MF, molecular function.

To find out which cellular functions and pathways the genes of the black module were involved in, GO and KEGG enrichment analysis were performed. GO analysis results indicated that the key genes of the black module mostly participated in the biological process (BP) of regulation of inflammatory response, and the main related molecular function (MF) terms were GTP binding, purine ribonucleoside binding, and purine nucleoside binding, but no cellular component (CC) was enriched (Figure 6A). Detailed information of GO enrichment was shown in Supplementary Tables S1, 2. KEGG analysis demonstrated that the most significantly enriched pathway was complement and coagulation cascades (Figure 6B).

### Validation of Key Genes in the GEPIA Database

**FIGURE 7:**
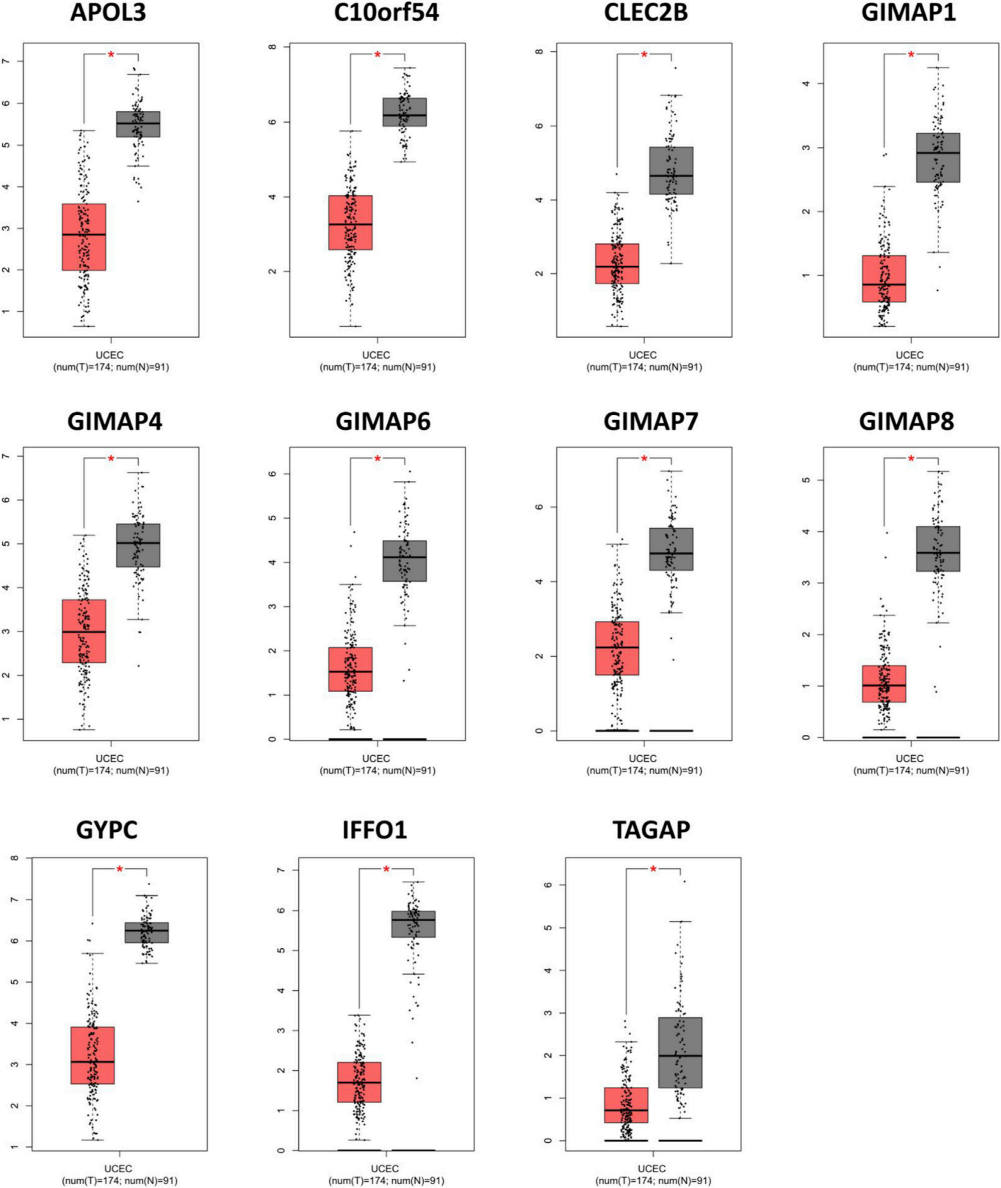
Validation of the abnormal expressions of the key genes *via* the online database of GEPIA. *p < 0.01.

Next, we verified the filtered key genes in the GEPIA database. The expression values of the 11 key genes were shown in Figure 7, indicating a significantly lower expression level in EC tissues compared with normal tissues for each key gene. In addition, we also showed the comparison of the expression levels of the 11 key genes in EC and normal tissues from five subsets of the TCGA-UCEC with boxplots (Supplementary Figure S3), and as we predicted, these genes were significantly lower-expressed in EC.

### Correlation Analysis of the Key Genes

**FIGURE 8:**
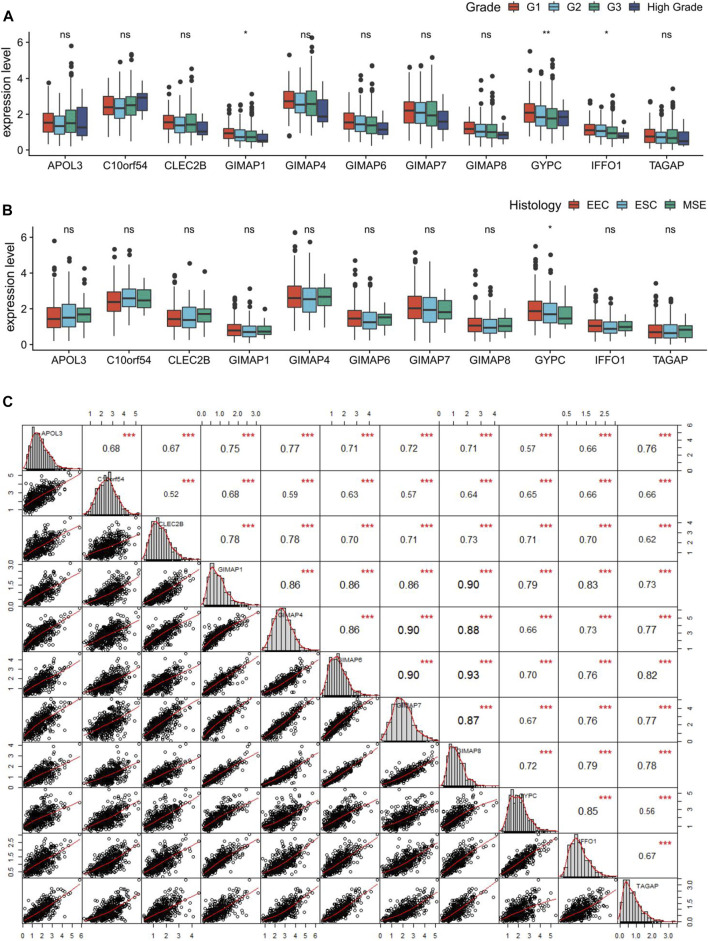
Correlation analysis of the key genes in endometrial cancer (EC). **(A**, **B)** Boxplots showing the correlations of the key genes with tumor grade and histology in EC. **(C)** Pearson correlation matrices between expression values of the key genes. *p < 0.05, **p < 0.01, **p < 0.001, and ns indicates no significance.

Given that immune score was associated with the tumor grade and histology in EC, we further compared the expression levels of the 11 key genes in different grades and different histological subtypes of EC. Differential analysis revealed significantly decreased expressions of GIMAP1, GYPC, and IFFO1 in high grade of EC (Figure 8A), and only GYPC was found to be correlated with different histological subtypes of EC (Figure 8B). Moreover, Pearson correlation analysis coupled with statistical significance demonstrated strong correlations between the expression values of these key genes, denoting their tight connections with each other. As the matrices shown, the minimum correlation coefficient among these genes was 0.52 while the maximum was 0.93. (Figure 8C).

### Survival Analysis

**FIGURE 9:**
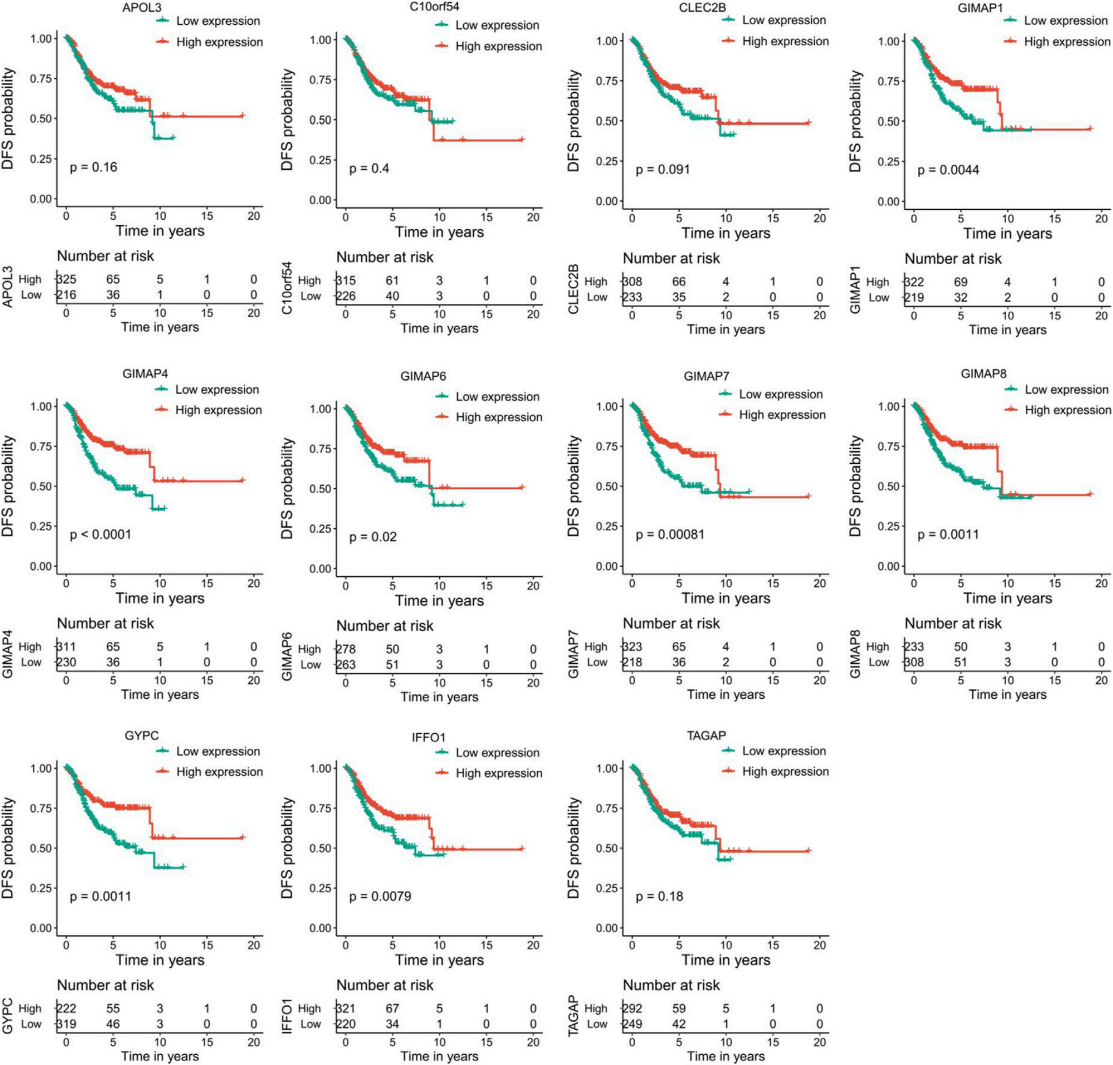
Kaplan–Meier survival curves of the 11 key genes characterizing DFS difference with log-rank tests in endometrial cancer. DFS, disease-free survival.

**FIGURE 10:**
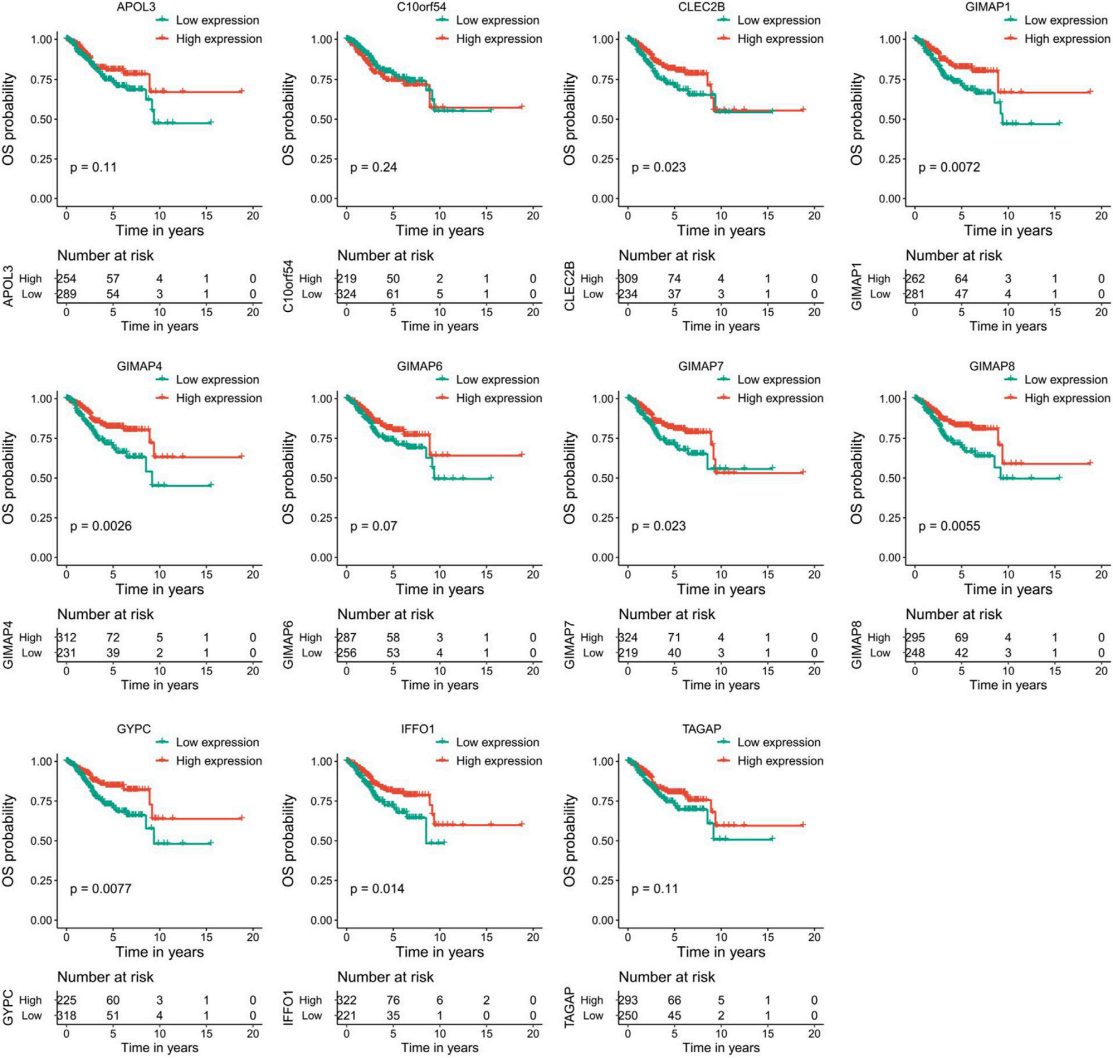
Kaplan–Meier survival curves of the 11 key genes characterizing OS difference with log-rank tests in endometrial cancer. OS, overall survival.

To evaluate the prognostic powers of these key genes, we examined the 11 key genes in perspective of DFS using Kaplan-Meier analysis and log-rank tests. We found that low expression of GIMAP1 (*p* = 0.0044), GIMAP4 (*p* = 0.0001), GIMAP6 (*p* = 0.02), GIMAP7 (*p* = 0,00,081), GIMAP8 (*p* = 0.0011), GYPC (*p* = 0.0011) and IFFO1 (*p* = 0.0079) were significantly associated with worse prognosis (Figure 9). For OS survival analysis, CLEC2B (*p* = 0.023), GIMAP1 (*p* = 0.0072), GIMAP4 (*p* = 0.0026), GIMAP7 (*p* = 0.023), GIMAP8 (*p* = 0.0055), GYPC (*p* = 0.0077) and IFFO1 (*p* = 0.014)) were found to be significantly associated with OS of EC patients (Figure 10).

### The key Genes were Significantly Associated with Infiltration of Immune Cells in EC Microenvironment.

**FIGURE 11:**
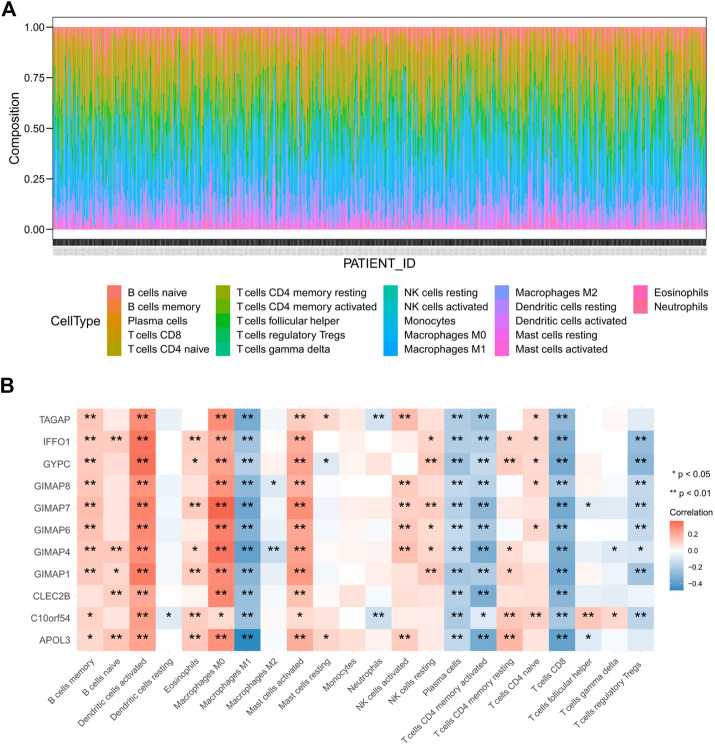
Immune infiltration cell profile for EC **(A)** and correlation between immune cell proportion and expression levels of key genes **(B)**.

To further explore the correlations of between the key genes’ expression levels and immune microenvironment, the proportions of 22 distinct immune cell types in EC samples were estimated with the CIBERSORT algorithm (Figure 11A). Next, we explored the correlation between immune cell type proportions and the expression levels of the 11 key genes, and the results indicated that all of these 11 key genes were significantly associated with at least eight kinds of immune cells (Figure 11B). All of the 11 key genes were positively correlated to the infiltration of activated Dendritic cells, M0 macrophages and activated mast cells, while they were all negatively correlated to the infiltration of M1 macrophages, plasma cells, activated CD4 memory T cells and CD8 T cells in EC samples. These results suggested that these key genes might be potential indicators for immune activity of IME.

## Discussions

In the United States, it was reported that 65,620 new cases and 12,590 deaths of EC occurred in 2020. The mortality has increased by approximately 1.4% per year from 2005 to 2014 (Torre et al., 2015; Siegel et al., 2020). The prognosis prediction of EC is largely based on histologic grade and clinical stage (Torre et al., 2015). Although several reports have pointed out the presence of immune dysregulation in EC and considered immune checkpoint blockade therapy as a potential treatment for EC patients (Le et al., 2015), the mechanism of the dysregulation of the IME in EC has not been completely revealed. In this study, we estimated the immune score for more than 500 EC samples and found that the immune score was significantly correlated with the grade and histology of EC. More importantly, it was strongly correlated with the OS and DFS of EC. For further study, we aimed at figuring out the key genes playing pivotal roles in the constitution of the immune microenvironment in EC.

Through subset-based analysis using the TCGA-UCEC dataset, we identified 758 upregulated genes and 1,179 downregulated genes in EC compared with normal samples. Further, we identified the black gene module that mostly correlated with immune score by WGCNA, which contained 71 differential immune-associated DEGs. Functional analysis revealed the module was mainly associated with the inflammatory response. Interestingly, five members of GTPase of immunity-associated protein (GIMAP) family (also known as immune-associated nucleotide-binding protein (IAN)) genes were included in the key genes. Numerous studies have reported that the function of the GIMAP family in regulating T cell development, selection, and homeostasis (Krucken et al., 2005; Nitta et al., 2006; Schnell et al., 2006). Therefore, it suggested that the five GIMAP family genes might play an important part in regulating the immune microenvironment in EC. In addition, two chemokines, CXCL10/IP-10 and CCL18 were unearthed. CXCL10 can recruit NK cells to tumor site and activate NK cells to kill cancer cells (Nagarsheth et al., 2017), and it has been reported strongly produced in tumor compared with the adjacent tissue in EC (Degos et al., 2019). CCL18, mainly secreted by tumor-associated macrophages (TAMs) in tumors, was positively correlated with malignancy in EC (Sakane et al., 2014; Jing et al., 2019). Our results verified that CXCL10 and CCL18 were two important factors in constituting the immune microenvironment of EC.

Through WGCNA analysis, we chose 11 key genes in the black module that were mostly correlated with immune scores for further investigation. In this study, the expressions of these key genes were significantly decreased in EC samples, which were confirmed by GEPIA database. Besides the five GIMAP family genes, TAGAP (T cell activation Rho GTPase activating protein) is also a GTPase related gene and an indicator of lymphocyte activation (Mao et al., 2004; Arshad et al., 2018). These abnormally expressed genes implied the immune disorders in the endometrial tumor environment.

Considering the correlation between immune score and clinical characteristics (grade and histology), we further quantified the correlations between the key genes and the tumor grade and histology in EC, individually. The expression levels of GIMAP1 and GYPC were significantly correlated to the tumor grade and only GYPC was significantly correlated to the tumor histology in EC. In the perspective of tumor grade, GIMAP1 showed the lowest expression in high grade while GYPC showed the lowest expression in G3. In addition, GYPC showed the lowest expression in MSE in the perspective of the histology of EC. Several studies reported that GIMAP1 is critical for the development of mature lymphocytes (Saunders et al., 2010; Webb et al., 2016; Datta et al., 2017). We observed that higher tumor grade of EC was accompanied by lower GIMAP1 expression and it seemed that higher grade of EC might have less functional lymphocytes infiltration in EC tumor microenvironment and led to a worse prognosis. Furthermore, the survival analysis verified that low expression of GIMAP1 led to a worse prognosis in EC. At last, all of the key genes were significantly and positively correlated with each other.

Given that the key genes were downregulated in EC samples, we explored whether their expression levels were correlated to DFS and OS. As a result, eight key genes (CLEC2B, GIMAP1, GIMAP4, GIMAP6, GIMAP7, GIMAP8, GYPC, and IFFO1) were positively correlated to the favorable prognosis. Interestingly, five members (GIMAP1, GIMAP4, GIMAP6, GIMAP7, GIMAP8) and four members (GIMAP1, GIMAP4, GIMAP7, and GIMAP8) of the GIMAP family were positively correlated with the DFS and OS, respectively. Combined with the results above, GIMAP family might hold great promise in EC exploration and deserve more attention in the future. As expected, GYPC was also related to the DFS and OS, suggesting its prognostic potential in EC. Besides, few Studies have been conducted in terms of IFFO1, the nucleoskeleton protein, which was recruited to the sites of DNA damage to promote the repair of DNA double-strand breaks (Li et al., 2019). Moreover, CLEC2B/AICL was reported as an important gene in NK cells stimulating NK cell effector function such as cytotoxicity and cytokine secretion (Neuss et al., 2018).

Previous studies have reported that the immune microenvironment was associated with the prognosis in EC (Ino et al., 2008; de Jong et al., 2009; Versluis et al., 2017). Therefore, we used CIBERSORT to estimate the infiltration of 22 immune cell types in EC samples. Preliminary results showed that macrophages and T cells might be the predominant immune cell types infiltrated in EC tumor microenvironment. Moreover, the key genes we found were strongly associated with multiple immune cell types infiltrated in EC tumor. The positive or negative correlation between the genes and immune cell types suggested lower or higher infiltration of those immune cell types in EC samples compared with normal samples. Obviously, there might be more M1 macrophages and CD8 T cells and less M0 macrophages and activated dendritic cells infiltrated in EC samples than those in normal samples.

However, limitations that should be mentioned: First, although we adopted the subset-based approach to balance the samples from the TCGA database, due to the small sample size of normal controls, more verifications are still required to enhance the robustness of the screened DEGs. Secondly, future biological experiments of *in vitro* and *in vivo* studies are needed to test and explore the effects of the key genes in EC.

Taken together, we discovered 11 key genes in this study, which were closely correlated with each other, abnormally expressed and associated with immune scores and clinical survival outcome in EC. They might play a critical role in the dynamic modulation of EC immune microenvironment and deserve further research in the future to reveal the specific functions and mechanisms in regulating immune cells infiltration or EC. A deeper understanding of these genes will throw light on the discovery of potential antibodies or small molecules for targeted therapy of EC by using effective computational methods such as network pharmacology and molecular docking.

## Data Availability

The data achieved and analyzed in the current study are available in the TCGA repository, https://portal.gdc.cancer.gov/.
