# Supplementary material for: Mining TCGA Data for Key Biomarkers Related to Immune Microenvironment in Endometrial cancer by Immune Score and Weighted Correlation Network Analysis
Source: Front Mol Biosci. 2021 Mar 26;8:645388. doi: 10.3389/fmolb.2021.645388 (PMC8048410; doi:10.3389/fmolb.2021.645388)
Supplement: Supplementary file 1 [file table1.docx]

Supplementary Material

## Supplementary Figures


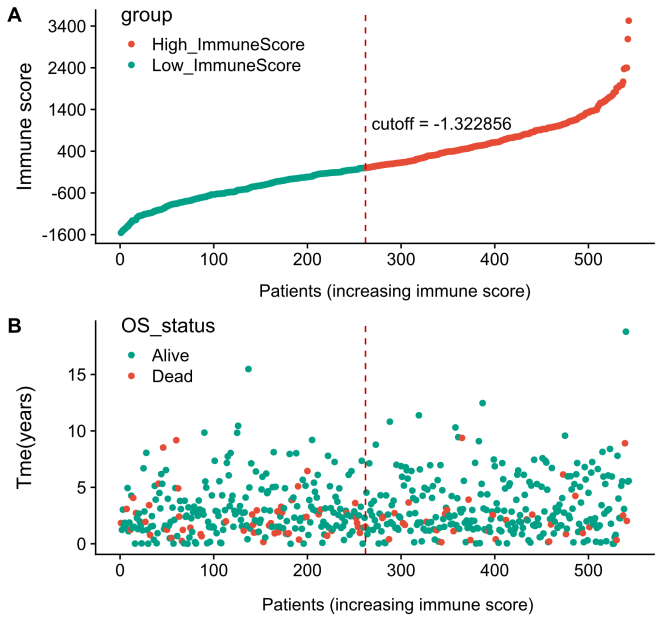

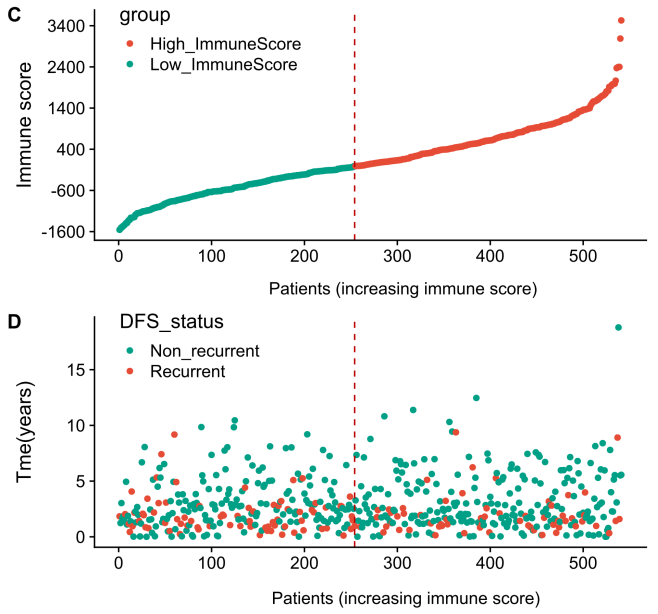


**Supplementary Figure 1 Correlation of immune score and patient survival outcome of endometrial carcinoma.** (A)-(B) Immune score and overall survival status for each patient. (C)-(D) Immune score and disease free survival status for each patient.


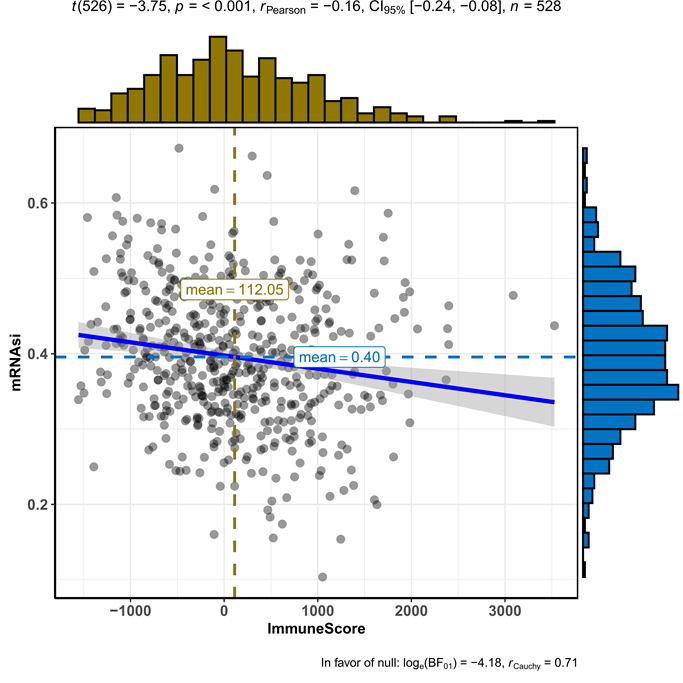


**Supplementary Figure 2** **Pearson correlation analysis between the mRNA stemness index and immune score. The blue line in the plot indicate the ﬁtted linear model.**


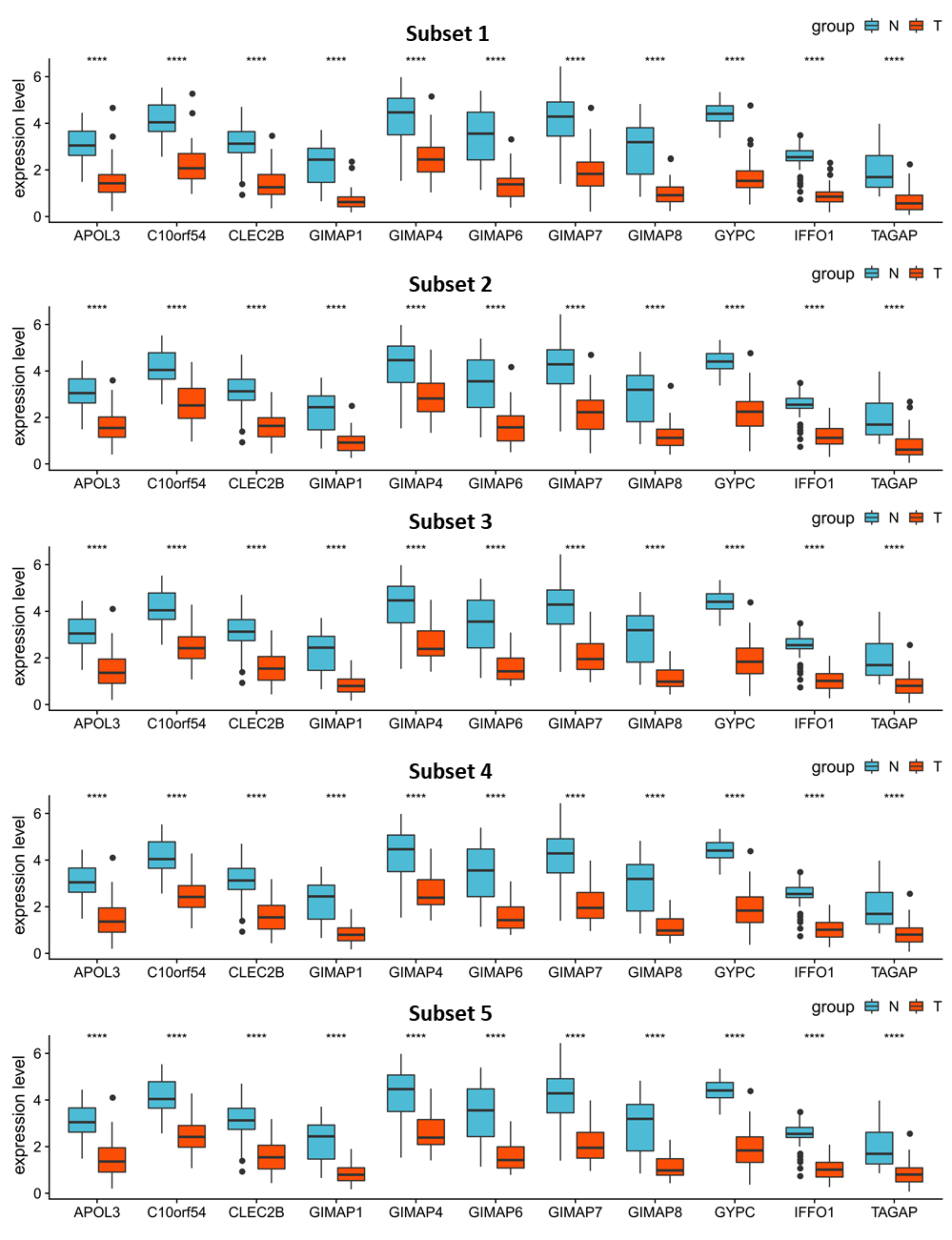


**Supplementary Figure 3 Comparison of the expression levels of the 11 key genes related to immune score from the five subsets of the TCGA-UCEC dataset. **** *P* < 0.0001.**
